# Exploiting the Cone of Influence for Improving the Performance of Wavelet Transform-Based Models for ERP/EEG Classification

**DOI:** 10.3390/brainsci13010021

**Published:** 2022-12-22

**Authors:** Xiaoqian Chen, Resh S. Gupta, Lalit Gupta

**Affiliations:** 1 School of Electrical, Computer, and Biomedical Engineering, Southern Illinois University, Carbondale, IL 62901, USA; 2 Center of Excellence for Stress and Mental Health, VA San Diego Healthcare System, San Diego, CA 92161, USA

**Keywords:** event-related potentials, electroencephalography, continuous wavelet transform, scalogram, cone of influence, subsample averaging, customized classifier design, channel ranking

## Abstract

Features extracted from the wavelet transform coefficient matrix are widely used in the design of machine learning models to classify event-related potential (ERP) and electroencephalography (EEG) signals in a wide range of brain activity research and clinical studies. This novel study is aimed at dramatically improving the performance of such wavelet-based classifiers by exploiting information offered by the cone of influence (COI) of the continuous wavelet transform (CWT). The COI is a boundary that is superimposed on the wavelet scalogram to delineate the coefficients that are accurate from those that are inaccurate due to edge effects. The features derived from the inaccurate coefficients are, therefore, unreliable. In this study, it is hypothesized that the classifier performance would improve if unreliable features, which are outside the COI, are zeroed out, and the performance would improve even further if those features are cropped out completely. The entire, zeroed out, and cropped scalograms are referred to as the “same” (S)-scalogram, “zeroed out” (Z)-scalogram, and the “valid” (V)-scalogram, respectively. The strategy to validate the hypotheses is to formulate three classification approaches in which the feature vectors are extracted from the (a) S-scalogram in the standard manner, (b) Z-scalogram, and (c) V-scalogram. A subsampling strategy is developed to generate small-sample ERP ensembles to enable customized classifier design for single subjects, and a strategy is developed to select a subset of channels from multiple ERP channels. The three scalogram approaches are implemented using support vector machines, random forests, k-nearest neighbor, multilayer perceptron neural networks, and deep learning convolution neural networks. In order to validate the performance hypotheses, experiments are designed to classify the multi-channel ERPs of five subjects engaged in distinguishing between synonymous and non-synonymous word pairs. The results confirm that the classifiers using the Z-scalogram features outperform those using the S-scalogram features, and the classifiers using the V-scalogram features outperform those using the Z-scalogram features. Most importantly, the relative improvement of the V-scalogram classifiers over the standard S-scalogram classifiers is dramatic. Additionally, enabling the design of customized classifiers for individual subjects is an important contribution to ERP/EEG-based studies and diagnoses of patient-specific disorders.

## 1. Introduction

The continuous wavelet transform (CWT) and the discrete wavelet transform (DWT) are indispensable signal processing tools that are extensively used to analyze and develop classifiers for event-related potential (ERP) and electroencephalography (EEG) signals. Like most practical signals, ERPs and EEG epochs have finite lengths. The CWT and DWT of finite-length signals suffer from border distortions which are called cone of influence (COI) effects and border effects, respectively. This study focuses primarily on emphasizing the importance of taking the COI into account in the design of ERP/EEG machine learning classification models which use features derived from the CWT. The COI is a boundary that is superimposed on the CWT scalogram to delineate the accurately computed wavelet coefficients from those that are inaccurate (artifact coefficients) due to the wavelet extending beyond the observation interval of the signal [1,2,3,4]. These edge-effect artifacts are due to the mechanics of convolving the signal and the wavelet near the beginning and near the end of the signal. For a given wavelet, the number of artifact coefficients computed depends on the wavelet scale. Larger scales result in a larger number of artifact coefficients; consequently, the boundary takes on a cone shape in the scalogram. Although it is common practice to exclude unreliable artifact coefficients outside the COI in scalogram analyses, surprisingly, the same cannot be said about classification. This is especially true when wavelet scalograms are used in conjunction with deep learning convolution networks (CNNs). Many recent studies have shown that the conversion of one-dimensional time-domain signals such as EEGs and ERPs into a time-scale matrix representation through the CWT not only offers simultaneous time-scale information but also offers an ideal matrix input format for CNNs [5,6,7,8,9,10,11]. However, due to the mechanics of matrix convolutions in the CNNs, the convolutions are carried out over the entire scalogram in the first stage as well as the subsequent convolution stages. Features extracted from the scalogram are also combined into feature vectors for input to many other classifiers such as support vector machines (SVMs) [7,12,13,14,15], random forests (RFs) [16,17], k-nearest neighbor (k-NN) [15,18,19], and multilayer perceptron (MLP) neural networks [20].

Considering the widespread use of the CWT for classifying ERPs and EEGs as well as numerous other types of non-stationary signals and the fact that COI edge-effect artifacts are well known, it is remarkable how little attention has been paid to addressing the following question: how can knowledge of the COI contribute to the design of wavelet-based classifiers? Our specific goal, therefore, is to demonstrate the importance of exploiting the information provided by the COI by developing three classification approaches that employ features from the (a) entire scalogram, (b) entire scalogram but with zeroed-out coefficients outside the COI, and (c) cropped scalogram consisting of only coefficients inside the COI. It will be shown that the entire scalogram is obtained via “same” convolution and the cropped scalogram is obtained via “valid” convolution; therefore, the entire and cropped scalograms will be referred to as the S-scalogram and V-scalogram, respectively. The scalogram with the zeroed-out coefficients will be referred to as the Z-scalogram. The S-scalogram approach is the “standard” method employed in most wavelet classifiers and can, therefore, serve as a baseline for performance comparisons with the two other approaches. We hypothesize that the Z-scalogram-based classifiers will outperform the S-scalogram-based classifiers, and the V-scalogram-based classifiers will outperform the Z-scalogram-based classifiers. Most importantly, we hypothesize that the improvement of V-scalogram classifiers over the standard baseline S-scalogram classifiers will be dramatic.

The formulations of the scalogram-based classifiers are quite general and are not tied to any particular application. This study focuses on ERP classification; however, the methods developed are equally applicable to classifying EEGs and other non-stationary signals. ERPs are voltage fluctuations in the EEG that occur as a result of an external or internal event and are frequently used to assess sensory, cognitive, motor, and emotion-related processes [21]. EEG is a non-invasive method used to assess neurophysiological function. EEG measures the electrical activity of large, synchronously firing populations of neurons in the brain with electrodes placed on the scalp [20]. EEG signals are often contaminated by various sources of noise, which can be physiological (e.g., eye blinks, eye movements, muscle artifacts) and non-physiological (e.g., electrode movement relative to the scalp, electrode wire movement, electromagnetic noise) in nature. Thus, preprocessing steps, including filtering, artifact correction, and artifact rejection procedures, are typically applied to the raw data [22]. Several other useful and novel methods have been proposed to reduce these sources of noise [23,24]. Altogether, EEG signals have been used in a wide variety of studies ranging from emotion classification [25] to qualifying seizure dynamics [26].

In order to validate the hypotheses and to also show that the performance trends are consistent across classifiers, the three approaches are implemented using a diverse set of classifiers which are trained and tested on the 64-channel ERPs of 5 subjects engaged in a binary semantic task which involved distinguishing between synonymous and non-synonymous word pairs. The problems with developing customized ERP classifiers for individual subjects are identified, and a subsampling strategy is developed to generate small-sample average ensembles to facilitate the design. A rank-of-rank sum channel selection strategy which uses an interclass separation measure is developed to select a subset of channels that carry the most useful discriminatory information for classifying the synonymous and non-synonymous ERPs. Classification experiments are designed using the (5 subjects) (8 channels) = 40 data sets, and it is shown that:

(a) the Z-scalogram classifiers outperform the standard S-scalogram classifiers;
(b) the V-scalogram classifiers significantly outperform the S-scalogram and Z-scalogram classifiers;
(c) the relative improvement of the V-scalogram classifiers over the standard S-scalogram classifiers is dramatic;
(d) the improvement trends across the three scalogram approaches are remarkably consistent across the classifiers, ERP channels, and subjects;
(e) the region outside the COI does not carry useful discriminatory information;
(f) the subsampling strategy to generate small-sample ERP ensembles enables customized classifier design for single subjects.

To the best of our knowledge, we are not aware of any other study that systematically demonstrates how the performance of standard scalogram-based classifiers can be improved through the V-scalogram approach, which excludes the artifact coefficients outside the COI. Furthermore, the subsampling strategy, which enables the design of customized classifiers for individual subjects, is unique.

## 2. Materials and Methods

This section presents (a) the definitions of the COI, S-scalogram, Z-scalogram, V-scalogram, and V-complement scalogram, (b) methods to reduce effects of edge artifacts, (c) measures to indicate the quality of scalograms, (d) the development of the S-scalogram, Z-scalogram, and V-scalogram approaches, (e) the subsampling strategy to generate small-sample ERPs to enable the development of ERP classifiers for single subjects, (f) the development of the algorithm to rank and select channels for classification problems, (g) the diverse set of classifiers selected to implement the 3 scalogram approaches for ERP classification, (h) the 64-channel dichotomous EEG data of 5 subjects that is used to implement the various classifiers for the three scalogram approaches, (i) the experiments designed to evaluate the performances of the various implementations of the three classification approaches, and (j) details of the wavelet choice, averaging parameter selection, feature vector generation, classifier architectures and hyperparameters, and cross-validation.

### 2.1. COI

The primary focus of this study is on the development of classifiers that modify the scalogram for feature extraction based on the COI. It is, therefore, important to understand how the COI is generated in the scalogram, which, in turn, requires an understanding of how the scalogram is generated from the CWT. Given a wavelet basis function $\Psi \left(t\right)$, the CWT of a signal $x\left(t\right)$ is given by

(1) $$W\left(a,b\right)=\frac{1}{\sqrt{a}}\int_{-\infty }^{\infty }x\left(t\right)\Psi^{*}\left(\frac{t-b}{a}\right)dt$$

where a and b are the scale and translation parameters, respectively. That is, the CWT is the convolution of $x\left(t\right)$ and scaled versions of $\Psi \left(t\right)$. Applying the CWT to $x\left(t\right)$ results in a set of wavelet coefficients which are a function of scale and position. A plot of the coefficients provides a time-scale representation of $x\left(t\right)$. In order to implement on a computer, the CWT is discretized; consequently, $W\left(a,b\right)$ is discrete both in scale and in time. The discretized CWT is often referred to as the DCWT to distinguish it from the discrete wavelet transform (DWT). Using s and n to denote the discrete-scale and discrete-translation variables, respectively, $W\left(s,n\right)$ plotted as a function of s and n is called the CWT coefficient matrix. The scalogram is the amplitude $\left|W\left(s,n\right)\right|$ or power ${\left|W\left(s,n\right)\right|}^{2}$ which can be represented by a matrix $G\left(s,n\right),\;s=0,1,\ldots \left(J-1\right);n=0,1,\ldots ,\left(N-1\right)$, where J is the number of scale bands, and N is the signal duration. In this zero-indexed matrix representation, row 0 corresponds to scale $S_{0}$, row 1 corresponds to scale $S_{1}$, …, row $\left(J-1\right)$ corresponds to scale $S_{J-1}$, with $S_{j}<S_{j+1}$.

**Figure 1:**
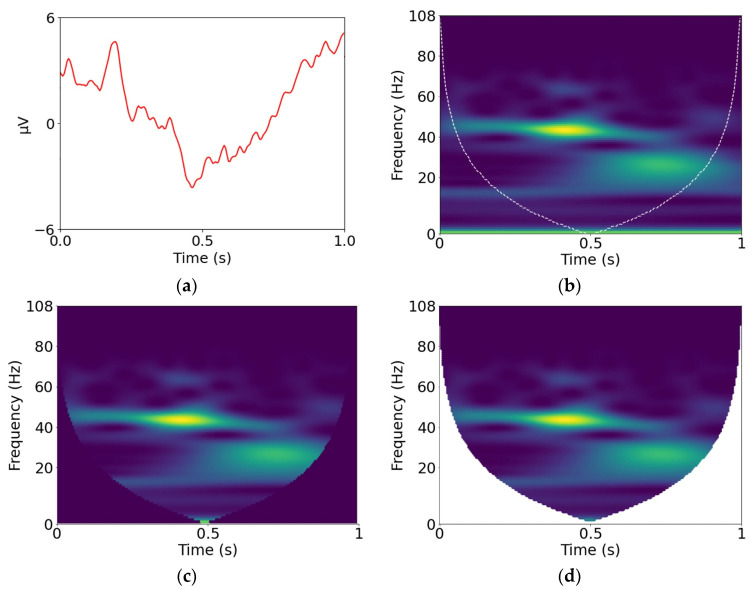
(**a**) ERP, (**b**) S-scalogram with superimposed COI, (**c**) Z-scalogram, (**d**) V-scalogram.

The COI is best explained by first understanding the convolution operations that are used to generate the coefficient matrix and to identify the COI. Each row of the coefficient matrix $W\left(s,n\right)$ is obtained from the linear convolution of $x\left(n\right)$ with a wavelet of a particular scale. There are several different forms of linear convolution, which include “full”, “same” and “valid”, which are useful for identifying the COI in the coefficient matrix. The three types of convolutions, which are described in detail in Appendix A, differ in the manner in which the start and end points are computed. The standard scalogram, which we refer to as the S-scalogram, is generated by same-convolution. The S-scalogram contains artifact coefficients because it suffers from edge effects. The valid convolution operation yields coefficients that are accurate and is used to identify the COI in the coefficient matrix. The boundary that separates the artifact coefficients from the accurate coefficients defines the COI in the S-scalogram. We refer to the part of the S-scalogram that is inside the COI as the V-scalogram because it corresponds to the coefficients associated with valid convolutions. Furthermore, the entire S-scalogram with zeroed-out coefficients outside the COI is referred to as the Z-scalogram. Figure 1 shows an ERP and the 3 different types of scalograms generated from the Morlet wavelet transform of the ERP.

#### 2.1.1. Edge Artifacts

It is inevitable that the CWT applied to a finite duration signal will lead to edge artifacts in the standard S-scalogram due to the need for extending the signal through padding. The inaccuracies of the artifact coefficients are the highest at the edges and decrease towards the COI. Various notable methods have been made to reduce, but not remove, the effects of the artifacts by smoothing the extensions [27,28,29], forecasting the extensions [30] and modifying the wavelet transform instead of extending the signal [31,32]. The effects of several extension methods have also been compared in [33,34]. For least-squares wavelet analysis (LSWA), which is used to analyze non-stationary signals that are not sampled evenly, the stochastic confidence level surface acts like the COI but is based on degrees of freedom [35]. 

Methods have also been proposed to approximate the COI, which include (a) using the time constant 1/e to delineate the borders of the cone of influence at each scale [2,36,37], (b) defining the extent of the COI at each scale as the point where the wavelet transform magnitude decays to 2% of its peak value [38,39], (c) delineating the COI by adding and subtracting (1/2) the wavelet footprint at the beginning and end of the observation interval at each scale [37,40,41], and (d) using the wavelet time interval that encompasses 95% of the wavelet’s energy for Morse wavelets [42,43,44].

#### 2.1.2. Scalogram Quality

In order to analyze the oscillatory behavior in most physiological signals, the scalogram is represented in terms of the discrete frequency variable f, instead of the scale variable. The discrete-time and discrete-frequency S-scalogram can be represented by the matrix $G_{S}\left(f,n\right),\;f=0,1,\ldots ,\left(J-1\right)$, where J in this case is the number of discrete-frequency bands. The COI depends not only on the wavelet choice and frequencies but also on the signal duration. For a given scalogram, the ratio ρ of the number of V-scalogram coefficients to the total number of S-scalogram coefficients can be used as a measure of the quality of the scalogram. For a (J×N) scalogram, the ratio is given by

(2) $$\rho =\;(\sum_{f=0}^{J-1}N_{f})/\left[\left(J\right)\left(N\right)\right],$$

where $N_{f}$ is the number of coefficients in row f that are inside the COI. For a given scalogram, a quality ratio β can also be defined in terms of the power enclosed by the COI to the total power in the following manner:

(3) $$\beta =\frac{\sum_{f}\sum_{n}{\left|W\left(f,n\right)\right|}^{2}}{\sum_{f=0}^{J-1}\sum_{n=0}^{N-1}{\left|W\left(f,n\right)\right|}^{2}}\;,$$

where the summations in the numerator are taken for values of $\left(f,n\right)$ that fall inside the COI. Higher values of ρ and β indicate better scalogram quality.

### 2.2. The Three Scalogram Approaches

Our goal is to compare the performances of classifiers that employ S-scalogram, Z-scalogram, and V-scalogram features; therefore, the most straightforward way to demonstrate this goal is to design classifiers for each ERP channel. Clearly, the same goal can be demonstrated by designing classifiers that combine information from multiple ERP channels [45,46]. However, the performance will then also depend on the combination methods which could mislead the performance comparisons. Therefore, the assumption hereafter is that the classifiers are single-channel classifiers.

The strategy to demonstrate the influence of the COI on classification performance is to develop three classification approaches in which the feature vectors are derived from (a) the S-scalogram $G_{S}\left(f,n\right)$, (b) the Z-scalogram $G_{Z}\left(f,n\right)$, and (c) the V-scalogram $G_{V}\left(f,n\right)$. The scalogram $G_{S}\left(f,n\right)$ consists of the signal power at each time-frequency location. For classification problems, transforming a signal $x\left(n\right)$ into $G\left(f,n\right)\;$is regarded as a feature extraction process. Clearly, additional operations can be performed on the scalogram feature matrix to generate other features such as the mean band power, ratios of power in select bands, and those which are typically used in image classification, such as texture, entropies, higher-order statistical features, and PCA-derived features. However, the classification results would then depend on the choice of the features making it difficult to draw direct conclusions about the influence of the COI on classifier performance. Therefore, in order to avoid this problem, no further modifications of the scalogram features are employed in the development of the classifiers. That is, the features are simply the scalogram coefficients.

The most direct way to demonstrate the performance trends across the three classification approaches is to concatenate the rows of the scalogram into a feature vector. In the formulation of the three classification approaches, the region outside the COI is represented by $G_{O}\left(f,n\right)$ such that

(4) $$G_{S}\left(f,n\right)=G_{V}\left(f,n\right)\cup G_{O}\left(f,n\right)$$

#### 2.2.1. S-Scalogram Approach

The feature vector in this approach is formed by concatenating the rows of the $\left(J\times N\right)$ S-scalogram. If Δ and T represent the row concatenation and transpose operations, the $\left[\left(J\right)\left(N\right)\times 1\right]$ feature vector $G_{S}$ is given by

(5) $$G_{S}={\left[\Delta_{f=0}^{J-1}G_{S}\left(f,:\right)\right]}^{T}$$

where, $G_{S}\left(f,:\right)$ is row f in $G_{S}\left(f,n\right)$. The feature vector $G_{S}$ includes a combination of accurately computed and artifact features. It can, therefore, be expected that the performance will be impacted negatively by the inclusion of the artifact features.

#### 2.2.2. Z-Scalogram Approach

In this method, the S-scalogram is modified by multiplying it with a mask $M\left(f,n\right)$ such that the resulting scalogram has elements set to zero outside the COI and are unchanged inside the COI. That is, the zeroed-out S-scalogram is given by

(6) $$G_{Z}\left(f,n\right)=G_{S}\left(f,n\right)\times M\left(f,n\right)$$

where

(7) $$M\left(f,n\right)=\left\{\begin{array}{c} 1,\;\;\;all\;\left(f,n\right)\;\in G_{V}\left(f,n\right)\\ 0,\;\;\;all\;\left(f,n\right)\;\in \;\;G_{O}\left(f,n\right) \end{array}\right.$$

The feature vector $G_{Z}$, which has the same dimension $\left[\left(J\right)\left(N\right)\times 1\right]$ as $G_{S}$, is given by

(8) $$G_{Z}={\left[\Delta_{f=0}^{J-1}G_{Z}\left(f,:\right)\right]}^{T}$$

Although the dimension of the feature vector $G_{Z}$ is the same as $G_{S}$, it can be hypothesized that the performance would improve by zeroing out the unreliable features that are outside the COI. However, including features with zero values across all classes is completely uninformative, providing no discriminatory value. Furthermore, the unnecessarily large feature vector can lead to overfitting, which can negatively impact the performance.

#### 2.2.3. V-Scalogram Approach

In this method, the S-scalogram is cropped with a COI-shaped mask $V\left(f,n\right)$ which selects the features inside the COI and discards the features outside the COI. The resulting COI-shaped 2-dimensional V-scalogram $G_{V}\left(s,n\right)$ is given by

(9) $$G_{V}\left(f,n\right)=G_{S}\left(f,n\right)∎V\left(f,n\right)$$

where ∎ is used to denote the cropping operation, and the mask selects or discards features according to

(10) $$V\left(f,n\right)=\left\{\begin{array}{c} select,\;\;\;all\;\left(f,n\right)\;inside\;COI\\ discard,\;\;\;all\;\left(f,n\right)\;outside\;COI \end{array}\right.$$

The $\left[\left(J\right)\left(N_{V}\right)\times 1\right]$ feature vector $G_{V}$ is given by

(11) $$G_{V}={\left[\Delta_{f=0}^{J-1}G_{V}\left(f,:\right)\right]}^{T}$$

where, $N_{V}=\overset{J-1}{\underset{f=0}{\sum }}N_{f}$, and $N_{f}$ is the duration of the frequency band f inside the COI. Unlike the two previous cases, the durations of the frequency bands are not equal; consequently, the feature vector $G_{V}$ does not have the same dimension as $G_{S}$. By excluding the features outside the COI, the dimension $\left(J\right)\left(N_{V}\right)$ of $G_{V}$ will be smaller than the dimensions of $G_{S}$ and $G_{Z}$. Through the simultaneous exclusion of unreliable features included in the S-scalogram approach and the cropping out of irrelevant features included in the Z-scalogram approach, it can be hypothesized that classifiers using $G_{V}$ for the feature vector should outperform the classifiers using the feature vectors of the S-scalogram and Z-scalogram approaches.

#### 2.2.4. V-Complement Scalogram Classification

In order to erase any doubts about the importance of excluding the features outside the COI, classifiers were also developed using only the features outside the COI, that is, the features contained in $G_{O}\left(f,n\right)$. This approach, represented by the $\bar{V}$-scalogram, is referred to as the V-complement scalogram approach because the feature set is the complement of the V-scalogram feature set. If these features are truly unreliable, as claimed throughout this study, the classification accuracies of the $\bar{V}$-scalogram classifiers should be worse than those of the baseline S-scalogram classifiers. The V-complement approach is not considered to be 1 of the 3 scalogram classification approaches but is simply developed to demonstrate that the features outside the COI do not carry any useful discriminatory information.

### 2.3. Subsample ERPs for Classifier Design

In statistical analyses, an ERP is formed by averaging all the time-locked single trials collected from a channel. The averaging operation is performed independently on the single trials of each channel. That is, 1 ERP is obtained for each channel. A different approach is required for ERP classification because classifiers are designed using the data split into two mutually exclusive subsets: the training set needed to train the model and the test set needed to evaluate the model. The term “design” includes classifier training and testing. Designing customized classifiers for individual subjects, whether it is through direct ERP-based classification or feature-based ERP classification, is impossible if each ERP category is represented by a single ERP. Group-based classifiers would require a very large number of subjects to generate enough ERPs for training and testing, which, in many practical cases, may be impossible. Also, it is important to note that the performance of a classifier is highly dependent on the number of ERPs and the quality of the training set. A small training set would not be representative of the variations in the ERPs, which results in poor generalization. Furthermore, large training sets are required to overcome the “curse of dimensionality” often encountered in the design of parametric classifiers [47]. Poor-quality (poor SNR) training sets lead to poor learning, which, in turn, results in poor performance. 

The most obvious approach to increase the size of the training and test sets is to simply use single trials without averaging. However, high classification accuracies cannot be expected with single trial signals due to the poor SNR. Because the SNR improves through averaging [48,49,50], it can be expected that the classification accuracy will improve by averaging over a small number of single trials. The classification accuracy can be expected to increase even further by increasing the number of trials averaged. For convenience, the signal formed by averaging m single trials will be referred to as an m-ERP, and m will be referred to as the “averaging parameter”. Furthermore, the full-sample ERP formed by averaging all Ω single trials in an ensemble will be referred to as the Ω-ERP, and, for convenience, a single trial will be referred to as a 1-ERP. In order to form an m-ERP, the 1-ERP ensemble can be partitioned sequentially into blocks of size m and then averaging the 1-ERPs in each block. Alternatively, subsamples of size m single trials can be drawn randomly without replacement and averaged to form distinct m-ERPs. However, in both cases, the sizes of the m-ERP training and test sets, which are equal to the number of 1-ERPs in the respective sets divided by m, “shrink” as m increases, which leads to overfitting. The obvious solution to this problem is to increase the size of the single trial ensembles. However, collecting large ensembles of single trials to form a large ensemble of m-ERPs for training and testing is difficult in practice because subjects tend to lose focus on the task due to fatigue and lapses in concentration over long data collection sessions. Another alternative is to average m single trials drawn with replacement to generate large ensembles of m-ERPs. However, there is the risk of generating identical m-ERPs, which can result in singular covariance matrices. This could be problematic for parametric classifiers such as those with Gaussian discriminant functions, which require the computation of the inverse of the covariance matrix [47].

The real challenge, therefore, in designing ERP classifiers for many applications is to avoid having to repeat the stimulus presentation numerous times to be able to form high SNR m-ERPs, especially during real-time testing. To be practical, most applications require accurate classification of ERPs averaged over a small number of single trials. The goal, therefore, is to enable the design of practical ERP classifiers that can accurately classify ERPs averaged over a small number of single trials without having to collect a prohibitively large ensemble of 1-ERPs.

#### 2.3.1. Generation of m-ERPs through “m-Subsample Averaging”

In order to overcome the shrinking problem that accompanies sequential averaging and averaging random subsamples without replacement, we employ a subsampling and averaging strategy introduced and implemented in [45,51,52]. We will refer to this method of generating subsample ERPs as “m-Subsample Averaging (m-SA)”. For a given 1-ERP ensemble of size $\Omega_{0}$, the m-SA strategy can be summarized as follows:

(a) Draw a random subsample of 1-ERPs of size m, $m<\Omega_{0}$. The m 1-ERPs in the subsample are drawn without replacement;
(b) Average the m 1-ERPs in the subsample to obtain an m-ERP;
(c) Replace the m 1-ERPs of the subsample into the single trial ensemble;
(d) Repeat steps (a)–(c) q times to generate an ensemble of $\Omega_{q}$ m-ERPs. Each ensemble generation is referred to as a “run”;
(e) Repeat steps (a)–(d) Q times to yield Q runs, with each run containing an m-ERP ensemble of size $\Omega_{q}$.

In order to maintain mutual exclusivity between the training and test sets, the 1-ERP ensemble is first partitioned into training and test sets, and subsequently, m-ERPs are generated from each of these sets. Each run, therefore, begins with randomly partitioned 1-ERP training and test sets. The specified number of m-ERPs for the training and test sets set is generated from the 1-ERPs of the training and test sets, respectively. Consequently, none of the 1-ERPs used to form the m-ERPs of the training set are included in forming the m-ERPs of the test set.

The key point to note is that m-SA offers the flexibility of generating large ensembles of m-ERPs for classifier design. Most notably, the m-ERP ensembles can be generated from the 1-ERP ensembles of individual subjects, thus enabling the design of customized classifiers for individual subjects. Clearly, m-SA is equally applicable for generating large ensembles for multi-subject group-based classifier design. Increasing the amount of data for training in this fashion is a form of data augmentation that can be especially beneficial for improving the performance of data-driven classifiers such as deep learning CNNs. Also noteworthy is that the training set can be made up of m-ERPs with different values of *m* to increase the amount of data and the variability in the training set. An additional point to note is that the averaging parameter does not have to be the same for the training and test sets. That is, the classifier could be trained with m-ERPs and tested on n-ERPs. In practice, it is desirable to keep both m and n small in order to avoid having to collect a large number of single trials for training and testing, respectively. It is especially important to obtain high accuracies for small values of n so that trials do not have to be repeated many times during testing.

### 2.4. Rank-of-Rank-Sum (RRS) Channel Ranking

The primary reasons for channel selection are to extract a subset of channels that carry useful discriminatory information and to reduce the computational burden. For ERP analysis, temporospatial PCA can be used to identify channels and time windows that best capture effects elicited by stimuli [53,54]. Therefore, the classification performance can be expected to improve by using only the identified channels and time windows. However, the drawback of using such prior knowledge for channel selection with respect to multichannel ERP classifier design is that the information that a particular classifier uses to discriminate between the ERP categories may differ from the information identified by PCA which is aimed at seeking projections that best represent the data in a least-square sense. A different approach which is aimed at selecting channels that best discriminate between the ERP classes is required for classification problems. A common approach is to use a suitable criterion with backward elimination to develop greedy algorithms which start with the full set of channels and decrease the number of channels, one channel at a time, to determine a subset of channels that yield the best criterion score. References [55,56,57] review several notable channel selection methods that have been developed for EEG channel selection.

The rank-of-rank-sum (RRS) algorithm developed in this study is feature-blind and quite general because it is formulated to rank channels for classification problems involving multiple classes (polychotomous) and multiple subjects. Binary classification and single-subject cases are treated as special cases of the RRS algorithm. The channels are ranked according to the interclass separations of the ERPs generated at the channels. The interclass separation measure selected is a scatter-normalized measure of separation between two cluster means. In general, higher classification accuracies can be expected if clusters have higher interclass separations. However, the interclass separation, which is a pairwise measure, is not directly applicable for multi-channel and polychotomous ranking because it provides a separation measure only for a pair of channels and a pair of ERP classes. Moreover, it is not directly applicable to multiple subjects. To solve the first problem, channel ranking is obtained via the ranking of the sum of the pairwise rankings of the $U\left(U-1\right)/2$ pairs of ERP classes where U is the number of ERP classes. One approach to solving the second problem is to combine the B subjects into a single group. This approach, however, is sensitive to the inter-subject variabilities of the ERPs. Instead, the RRS algorithm solves the second problem by determining the rankings of the sum of the channel rankings across the B subjects. The RRS algorithm is described in detail in Appendix A.

### 2.5. Choice of Classifiers

In order to test the performance-related hypotheses and to determine whether the performance trends are consistent across classifiers, channels, and subjects, a range of diverse classifiers is selected to implement the three scalogram approaches for ERP classification. The following well-known classifiers are selected: SVMs, RFs, k-NN, MLPs, and deep learning CNNs. Each of these classifiers has its advantages and limitations. Details of these well-known classifiers can be found in excellent books on machine learning [58,59,60,61,62].

Two variants of CNN classifiers are designed: vector input CNN-1 and matrix input CNN-2. The CNN-1 classifier accepts vector inputs and can, therefore, be implemented for all 3 approaches through the row concatenation operations described in Section 2.2. As mentioned in the introduction, the matrix scalogram is an ideal format for CNN inputs. Implementing matrix input CNN classifiers for the first 2 approaches is straightforward because the S-scalogram and the Z-scalogram have matrix formats. However, implementing CNN classifiers capable of accepting the unequal row V-scalogram matrix is problematic because the CNN matrix convolutions require equal row matrices. Such an implementation is beyond the scope of this study.

### 2.6. Data Sets

The EEG data used to demonstrate the application and evaluate the performance of the 3 classification approaches was downloaded from:

https://eeglab.org/tutorials/10_Group_analysis/study_creation.html#description-of-the-5-subject-experiment-tutorial-data (accessed on 1 October 2022).

This data set was selected because it was freely available and ideal for demonstrating the goals of this study. However, the methods formulated in this study are quite general and are not tied to this particular data set or any other data set. According to the description of the study on the website, the EEG data were collected from 5 subjects performing an auditory binary semantic task in which they were asked to distinguish between synonymous and non-synonymous word pairs. The second word was presented 1 s after the presentation of the first word. Data epochs were extracted 2 s before the second-word onset to 2 s after the second-word onset. EEGs were collected from 64 channels using a sampling rate of 200 Hz. After decomposing each subject’s data by ICA, 2 EEG datasets were extracted: the synonymous data set comprising trials in which the second word was synonymous with the first one and the non-synonymous data set in which the second word was not a synonym of the first. Thus, the study included 5 binary data sets or (5 subjects) (2 conditions) = 10 datasets for each of the 5 subjects. Both datasets of each subject were recorded in a single session. The number of epochs for each condition varied from 196 to 235 across the 10 data sets. 

For the purpose of this study, the single trials (1-ERPs) for the classification experiments consisted of the 1 s segment extracted after the second-word onset in each epoch. Consequently, the total number of samples in each 1-ERP was 200. In order to facilitate 5-fold cross-validation and to have an equal number of epochs across the 10 data sets, the first 195 epochs were selected from each data set. The top 8 ranked channels for each subject determined by the RRS algorithm were selected for the experiments. The rankings are listed in Table 1. The rankings of the channels differ across the 5 subjects, which is not unexpected. Whether these channels differ from those extracted from temporospatial PCA is not of concern because the focus is on channel selection for classification and not for analyses. Furthermore, whether the top-ranked channels are correlated proximal channels is not taken into account because the goal is to simply select 1-ERPs from a subset of channels, instead of all channels, that can be used as data sets for the experiments. If needed, correlation analyses could be conducted on the ranked channels to systematically include low-correlated channels starting with the top-ranked channel.

**Figure 2:**
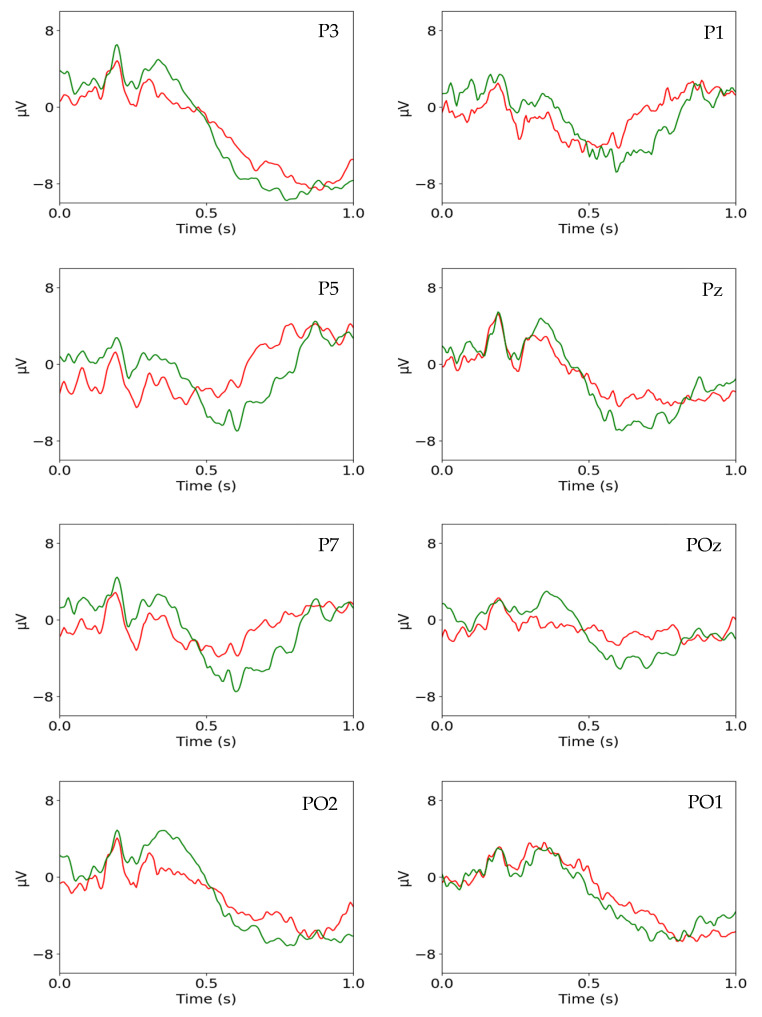
The full-sample synonymous (green) and non-synonymous (red) Ω-ERPs of the 8 selected channels of subject $b_{1}$.

Each classifier was designed on (5 subjects) (8 channels) = 40 binary data sets. The 40 binary data sets are large enough to adequately observe the performance trends across the three approaches. Adding more channels will simply increase the size of the already large tables that are included in the results without contributing to the trend analyses. Figure 2 shows the full-sample Ω-ERPs of subject $b_{1}$ formed by averaging the 1-ERPs in the binary data sets. Observe that the Ω-ERPs of the two classes appear to be fairly well separated in all 8 channels.

### 2.7. Classification Experiments

This section describes various aspects of the design of experiments to test the implementations of the three classification approaches using the 5 different types of classifiers and the 5-subject 8-channel ERP data sets.

#### 2.7.1. Wavelet Choice

Over the years, many different CWTs have been proposed, which include, among many others, Morlet [63,64], Morse [44], Gaussian [2], and Mexican Hat [65,66]. The choice of the wavelet is application dependent, and in this study, the analytic Morlet wavelet is selected because it is frequently used to analyze the oscillatory behavior of ERPs and EEGs. The analytic Morlet mother wavelet, a product of a complex exponential signal of frequency $f_{0}$ and a zero-mean Gaussian window with variance $\sigma^{2}$, is given by

(12) $$\Psi_{f_{0}}\left(t\right)=\left(A\right)(e^{-\frac{t^{2}}{2\sigma^{2}}})\left(e^{j\left(2\pi f_{0}\right)t}\right)$$

where the constant A ensures that the wavelet energy is equal to one. The analytic wavelet coefficients are complex, and the power spectrum is zero for negative frequencies. Note that the wavelet is defined in terms of a frequency parameter which is preferred in applications involving oscillatory analyses.

#### 2.7.2. Selection of the Averaging Parameter

The aim of this study is not focused on obtaining the highest classification accuracies but on analyzing the performance trends across the 3 classification approaches using the data sets. In general, classifiers trained and tested on high SNR data sets yield good accuracies across the classifiers, making it difficult to compare their performances. Furthermore, it may not be practical to repeat trials a large number of times to generate high-SNR m-ERPs during real-time testing. It is, therefore, important to be able to obtain high accuracies for small values of m. For these reasons, the smallest value, m=4, which enabled the analyses of the performance trends across the 3 approaches, was selected for generating the m-ERP ensembles. In the experiments to follow, a classifier was developed to test the 4-ERPs of each channel collected from each subject independently.

#### 2.7.3. Feature Vector Generation

The feature vectors for each approach were generated from the Morlet wavelet power scalogram through row concatenation, as described in Section 2.2. The question as to whether the scalogram is computed from 1-ERPs or m-ERPs has to be considered. In general, the scalogram of the average of the single trials is used for evoked power analysis, whereas the average of the scalograms of each single trial, which gives the total power, is used for induced power analysis. The total power is the sum of the evoked and induced power. In this study, for the purpose of feature extraction, a single scalogram is computed for each m-ERP where m is equal to 4. The duration of each 4-ERP (same as the single trial duration) was N = 200 samples, and the number of frequency bands J was 108. The dimensions of the scalograms were, therefore, 108 × 200. Hence, the dimension of the feature vectors $G_{S}$ and $G_{Z}$ were (21,600 × 1). Using the process outlined in Section 2.2 for selecting the features inside the COI, the dimension of the feature vector $G_{V}$ was 17,056 × 1. The ratio ρ of the number of V-scalogram coefficients to the total number of S-scalogram coefficients for this case is (17,056/21,600) (100) = 78.96%. That is, approximately 21% of the scalogram coefficients were artifact coefficients.

#### 2.7.4. Classifier Architectures and Parameters

This subsection describes the architectures and hyperparameters of the 6 different classifiers that were implemented. No serious attempt was made to optimize the architectures and hyperparameters because the goal is to compare the relative performances of a given classifier using the S-scalogram, Z-scalogram, and V-scalogram feature vectors. Details of the classifiers are as follows:

SVM Classifiers: The SVM classifiers were implemented using the Gaussian radial basis function kernel. The best combination of the regularization parameter C and influence parameter γ was selected using an exhaustive grid search. 

RF Classifiers: The following hyperparameters were selected: number of trees = 10, maximum depth of a tree = 475, minimum number of samples required to split an internal node = 3, minimum number of samples required to be leaf node = 1, splitting quality criterion = entropy.

k-NN Classifiers: The Euclidean distance metric was used to measure proximity. Through trial and error, it was found that k = 20 yielded good performance across all classifiers.

MLP Classifiers: The MLP classifiers consisted of four layers of neurons: the first layer had 1024 neurons, the second layer had 512 neurons, the third layer had 256 neurons, and the output layer had 2 neurons. The sigmoidal activation function was used in all 4 layers. The following training options were used: initialization = uniform random, optimizer = Adam, learning rate = 0.001, number of epochs = 50, drop-out probabilities = 0.15.

CNN-1 Classifier: The CNN-1 classifiers consisted of three convolution layers followed by a max pooling layer and terminated with a fully connected network (FCN) consisting of four layers of neurons. The convolution layers used the ReLU activation functions, the first three layers in the FCN used sigmoidal activations, and the last layer used softmax activations. The “same” operation was used in the convolution layers. The number of filters in the convolution layers was 32. The filter dimensions in the convolution layers were $\left(9\times 1\right)$. A (2×1) max pooling filter with a stride of 2 was used in the pooling layer. The FCN had 1024, 512, 256, and 2 neurons in the four layers. The following training options were used: initialization = uniform random, optimizer = Adam, learning rate = 0.001, number of epochs = 50, drop-out probabilities = 0.15.

CNN-2 Classifiers: The CNN-2 classifiers consisted of a convolution layer, max pooling layer, convolution layer, max pooling layer, convolution layer, max pooling layer, and terminated with a fully connected network (FCN) consisting of four layers of neurons. The convolution filters had dimensions $\left(3\times 3\right),$ and the max pooling filter had dimensions $\left(2\times 2\right)$ with a stride equal to 2. The “same” operation was used in both convolution layers. The number of filters in both convolution layers was 32. The convolution layers used the ReLU activation functions, the first three layers in the FCN used sigmoidal activations, and the last layer used softmax activations. The FCN had 1024, 512, 256, and 2 neurons in the four layers. The following training options were used: initialization = uniform random, optimizer = Adam, learning rate = 0.001, number of epochs = 50, drop-out probabilities = 0.15.

In summary, the SVM, RF, k-NN, MLP, and CNN-1 classifiers were developed for all three scalogram approaches, and the CNN-2 classifiers were developed for the first 2 approaches to give a total of 17 classifiers. Each classifier was trained and tested independently on the 40 binary data sets. The classifiers were implemented using the PyTorch library.

#### 2.7.5. Cross-Validation

The steps outlined in Section 2.3.1 were used to generate runs consisting of the m-ERPs of the training and test sets. For each run that started with randomly ordered 1-ERPs, the 195 1-ERPs of each class were partitioned into 5 folds, each containing 39 1-ERPs. The training set consisted of 4 folds (156 1-ERPs), and the test was the remaining fold. From the 1-ERP training set, an equal number (156) of 4-ERPs were generated for the training set. Similarly, 39 4-ERPs were generated for the test set. This method of generating 4-ERP training and test sets was conducted for each channel. The channel classifiers were trained and tested using 5-fold cross-validation for each run. For each run, the total number 4-ERPs for training and testing each channel classifier consisted of (195 4-ERPs/class) (2 classes) = 390 4-ERPs. The classification accuracy for each run, expressed as a percentage, was estimated as the ratio of the correctly classified 4-ERPs to the total number (390) of 4-ERPs of the 2 classes. The procedure was repeated over 50 runs, and the final classification accuracy of each classifier was estimated by averaging the accuracies of the 50 runs. That is, the final classification accuracy of each classifier was averaged across (390) (50) = 19,500 4-ERPs belonging to the 2 classes. Importantly, all the classifiers were implemented using identical training and test sets.

## 3. Results

This section presents the results of the various experiments designed to observe the performance trends of the 3 classification approaches across classifiers, channels, and individual subjects. 

### 3.1. m-ERP Classification Results

In order to observe the trends across the 3 approaches closely, the final classification accuracies obtained from the classifiers across the three approaches are presented separately in five “full” tables, which contain the results for each subject. Due to the large size, the five full tables are presented in Appendix B (Table A1, Table A2, Table A3, Table A4 and Table A5). Each entry in the tables is the average classification accuracy obtained from testing (390 4-ERPs × 50 runs) = 19,500 4-ERPs. The best result for each channel is highlighted in bold font. The entries are empty for the CNN-2 classifiers for the V-scalogram approach because they were not implemented. Any inconsistent result (expected improvement is not observed) is highlighted in red. Out of the total of 680 results presented in the five full tables, only 10 (<1.5%) were minorly inconsistent in the sense that the expected improvements of the Z-scalogram classifiers over the S-scalogram classifiers were not observed. Table 2 summarizes the overall average classification across the five subjects and eight channels for each approach. Except in the row labeled Global Average, each entry in Table 2 is the average classification accuracy obtained from testing (five subjects × eight channels × 19,500 4-ERPs) = 780,000 4-ERPs. The row labeled Global Average is the average of the classifiers across each approach (column averages). An asterisk on CNN-2 indicates that the CNN-2 results were not included in the computation of the Global Average. The CNN-2 averages are included below the Global Averages to emphasize this point.

**Figure 3:**
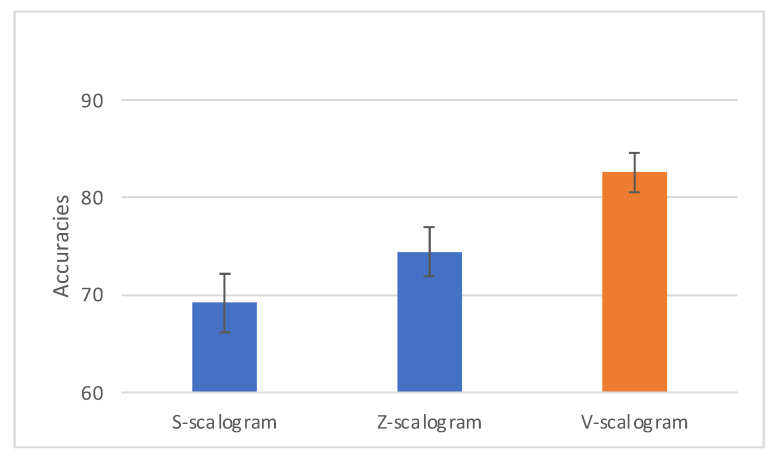
The 95% CIs for the global mean accuracies of the 3 approaches.

Figure 3 shows the 95% confidence interval error bars for the Global Averages in Table 2. The V-scalogram error bar does not overlap with the S-scalogram and Z-scalogram error bars. Therefore, the improvements of the V-scalogram approach over the S-scalogram and Z-scalogram approaches are statistically significant, with *p*-values less than 0.05.

### 3.2. Single Trial Results

Up to this point, we have assumed that single trial classification will yield poor classification accuracies. In order to support this assumption, the classifiers were trained and tested on the 1-ERPs of Subject $b_{3}$. The results are shown in Table A6 in Appendix B. For brevity, the results of the 4 other subjects are not included because the trends are the same as those observed for Subject $b_{3}$. As expected, the results for the 1-ERP classification are inferior to the 4-ERP classification. Interestingly, the CNN-1 results using V-scalogram features are quite good for single trial classification. The significant jumps in the CNN results from the S-scalogram accuracies to the V-Scalogram accuracies are noteworthy.

### 3.3. V-Complement Scalogram Results

Classifiers were developed using only the $\left(21600-17056\right)=4544$ features outside the COI. Table 3 summarizes the classification accuracies of the classifiers trained using this $\bar{V}$-scalogram approach. The S-scalogram results are also included in the table to facilitate comparison. The $\bar{V}$-scalogram average classification accuracy of 51.95% is close to the 50% accuracy expected for random binary classification. Clearly, the artifact coefficients do not carry useful discriminatory information. Therefore, the performance should improve by zeroing the artifact coefficients and improve even further by removing them completely, as confirmed by the experimental results in Appendix B.

## 4. Discussion

This section discusses the findings presented in the Section 3.

### 4.1. Performance Trends

The following general trends in the 6 full tables in Appendix B and the summary table are important to note: for each classifier, there is an improvement in the performance of the Z-scalogram classifiers over the S-scalogram classifiers (including CNN-2) and a consistent improvement of the V-scalogram classifiers over the Z-scalogram classifiers. This is also reflected across the overall averages for the three approaches. Importantly, the CNN-1 results in Table A1, Table A2, Table A3, Table A4 and Table A5 also show that the two-class semantic ERPs can be classified with accuracies exceeding 94% for all 5 subjects using a value as small as m = 4 for subsample averaging with the V-scalogram. As noted in the subsampling section, even higher accuracies can be obtained by increasing the averaging parameter m.

For the CNN-2 classifier, the improvement of the Z-scalogram approach over the S-scalogram approach is also notable. Quite interestingly, even the Z-scalogram approach can yield accuracies greater than 93% for all five subjects. This impressive performance can be attributed to the fact that the convolution filters are trained to extract discriminatory features from input during the training phase. That is, the zeroed-out region outside the COI is ignored to some degree by the filters. The filters are influenced by the zeroes to a greater extent when they overlap with scalogram regions that are both inside and outside the COI boundary. Based on the observed trends of the other classifiers across the three approaches and the trends between the two CNN-2 implementations across the first two approaches, we can expect a dramatic improvement in the CNN-2 implementation of the V-scalogram approach over the standard S-scalogram approach if the convolution mechanisms in the CNNs can be modified to accommodate unequal row matrices. Furthermore, through an understanding of how the filter weights are trained by convolving over entire matrices for S and Z-scalograms containing artifact and zeroed-out coefficients, respectively, it can be expected that the convolution filters trained only over the correct V-scalogram information are bound to lead to better performance. It is important to note that modifying wavelet-based CNN classifiers to restrict the matrix convolutions within the COI would be a major breakthrough, given the huge attention focused on developing and applying wavelet-based CNN classifiers to numerous problems in various fields. Future efforts will focus on developing direct/indirect methods to restrict matrix convolutions within the COI.

### 4.2. Relative Improvements

The consistent improvements of the Z-scalogram and V-scalogram approaches over the baseline S-scalogram approach are quite remarkable because they are observed across the m-ERPs of all 5 subjects across all 8 channels (all 40 data sets). The relative improvement in the performance of an approach *B* over an approach *A*, denoted by $\Gamma \left(B,A\right)$, can be measured using

(13) $$\Gamma \left(B,A\right)=\left[\frac{\alpha \left(B\right)-\alpha \left(A\right)}{\alpha \left(A\right)}\right]\times 100$$

where $\alpha \left(A\right)$ and $\alpha \left(B\right)$ are the classification accuracies of approaches A and B, respectively. Table 4 summarizes the relative improvements for the average accuracies listed in Table 2. These results clearly confirm that the Z-scalogram approach outperforms the S-scalogram approach and the V-scalogram approach outperforms the Z-scalogram approach. Most importantly, the improvements of the V-scalogram approach over the standard S-scalogram approach are dramatic.

### 4.3. Comparison of Classifiers

Although the primary goal of this study is to show that for any given classifier, the performance of the classifier using the standard S-scalogram features can be improved by using Z-scalogram features, and further improvements can be obtained by using V-scalogram features, the results in the tables can also be used to rank the 6 different classifiers to pick the best performing classifier type. From the averages in Table 2, it can be concluded that the best results for the V-scalogram approach across classifiers, channels, and individual subjects were obtained with the CNN-1 classifiers. The superiority of the CNN classifier models can be attributed to the fact that the filters in the convolution layer have the ability to extract complex features that are relevant to classification. Most importantly, the scalograms can be input directly to the CNNs without having to extract a set of hand-engineered features. Consequently, the performance is not dependent on the hand-engineered feature set, which is often determined through trial and error. The size and the shape of the convolution filters can couple information in different ways to extract different types of features from the same input presented to the CNN. For example, row filters couple information from individual frequency bands in the scalogram. A matrix filter can be used to couple information across frequency bands. If needed, the complexity of the features can be increased by increasing the number of convolution layers. In general, CNNs are highly scalable for larger classification problems by increasing the network depth and increasing training. Therefore, CNNs should be a good choice for multi-subject group-based classification. Furthermore, there are several elegant ways of fusing “information” from multiple channels with CNNs to improve performance. The information can be data/features of the channels or decisions of the channel classifiers. The study in [67] involving the classification of multi-axial (multi-channel) signals using CNNs shows that, in general, multi-axial fusion classification models outperform the best uniaxial (single-channel) classification model.

## 5. Conclusions

The goal of this study was to demonstrate the importance of exploiting information offered by the COI in designing CWT-based classifiers. It was hypothesized that the performance would improve by zeroing out the unreliable scalogram features outside the COI and that the performance would improve even further by completely excluding the unreliable features outside the COI. A subsampling strategy was developed to generate small-average ensembles to enable customized design for individual subjects/patients, and a rank-of-rank sum channel selection strategy was developed to select a subset of channels from multiple channels. The S-, Z-, and V-scalogram classification approaches developed to validate the performance hypotheses were implemented with a set of diverse classifiers and tested on semantic task ERPs of 5 subjects. The results from 40 data sets confirmed the expected relative improvements, and it was noted that the improvements were remarkably consistent across channels, classifiers, and subjects. Most importantly, the relative improvements of the V-scalogram classifiers over the standard S-scalogram classifiers were dramatic. Among the 5 classifier types implemented, the CNN classifiers yielded the best results consistently. 

In conclusion, this study demonstrated unequivocally that excluding the region outside the COI for feature extraction results in a significant improvement in classifier performance. The V-scalogram approach is not restricted to ERP/EEG classification and is applicable to improving the performance of wavelet classifiers designed to classify other physiological signals as well as signals in many other fields, including engineering, science, and economics. Undeniably, this is a significant contribution, considering the vast number of wavelet-based classifiers that have been reported but have not exploited the COI for feature extraction in the manner described in this study. Furthermore, the ability to design customized classifiers for individual subjects which yield high classification accuracies using subsample averaging is an important contribution for studying and diagnosing patient-specific disorders using ERPs and EEGs.

## Figures and Tables

**Table 1:** The top 8 ranked channels for each subject.

Subject	Top 8 Ranked Channels							
$b_{1}$ $b_{2}$	P3 PO4	P1 F3	P5 CP5	Pz PO2	P7 Oz	POz	PO2	PO1
						PO6	TP7	O1
$b_{3}$	O2	Oz	O1	PO6	PO4	PO2	TP7	POz
$b_{4}$	O2	Oz	TP7	O1	F3	F1	F5	CP5
$b_{5}$	C2	P5	Cz	C4	P3	P7	C1	FCz

**Table 2:** Average classification accuracies of the six different types of classifiers across the entire test set consisting of 780,000 4-ERPs. The global average is the average of the column entries in the first five rows of the table.

Classifier		Approach	
	S-Scalogram	Z-Scalogram	V-Scalogram
SVM	58.33	62.49	70.95
RF	75.30	81.74	89.07
k-NN	58.85	62.92	70.86
MLP	75.84	81.27	89.34
CNN-1	77.68	83.91	92.96
Global Average	69.20	74.46	82.64
CNN-2 *	81.18	92.73	-----

* indicates that the CNN-2 results were not included in the computation of the Global Average.

**Table 3:** A comparison of the average classification accuracies of the S-scalogram and the $\bar{V}$-scalogram approaches.

Classifier	Approach	
	S-Scalogram	$\bar{V}$-Scalogram
SVM	58.33	50.77
RF	75.30	51.92
k-NN	58.85	47.08
MLP	75.84	53.85
CNN-1	77.68	56.15
Global Average	69.20	51.95

**Table 4:** Relative improvements in the accuracies listed in Table 2.

Classifier	$\Gamma \left(Z,S\right)$	$\Gamma \left(V,Z\right)$	$\Gamma \left(V,S\right)$
SVM	7.13	13.54	21.64
RF	8.55	8.97	18.28
k-NN	6.93	12.62	20.42
MLP	7.15	9.94	17.80
CNN-1	8.02	10.80	19.68
Global Average	7.61	10.97	19.42
CNN-2	14.24	-----	-----

## Data Availability

Not applicable.

## Appendix A

The convolution operations that lead to the definitions of the S-scalogram, Z-scalogram, V-scalogram, and COI are described in detail in this Appendix. The formulation of the generalized RRS channel selection algorithm applicable to single/multiple subjects and dichotomous/polychotomous classification is also presented in this Appendix.

### Appendix A.1. Types of Convolution

The question that is addressed in this section is: what type of convolution causes the edge artifacts to occur in the scalogram coefficient matrix? In order to answer this question, three convolution mechanisms are described by assuming the durations of the signal $x\left(n\right)$ and the zero-centered mother wavelet filter $h\left(n\right)$ are N and $\left(2M+1\right)$, respectively. That is, the compact support of $h\left(n\right)$ is $\left[-M,M\right]$. It is also assumed that M<N.

#### Appendix A.1.1. Full-Convolution

In general, the discrete convolution sum is given by

(A1) $$y\left(n\right)=\sum_{\lambda =-M}^{M}x\left(\lambda \right)h\left(n-\lambda \right)$$

where λ is a dummy variable. That is, the convolution operation is performed by shifting a folded version of $h\left(\lambda \right)$ along $x\left(\lambda \right)$ and finding the dot product at each shift n. It is assumed that the shift occurs in unit steps. In order to simplify the discussion, it will be assumed that $h\left(n\right)$ is symmetric. In full convolution, the rightmost sample of $h\left(\lambda \right)$ is aligned with the leftmost sample of $x\left(\lambda \right)$ at the start of convolution, and the leftmost sample of $h\left(\lambda \right)$ is aligned with the rightmost sample of $x\left(\lambda \right)$ at the end of convolution. By convention, the filtered output is at the filter center; therefore, the duration of the filtered output is $\tilde{N}=\left[N+\left(2M+1\right)-1=N+2M\right]$. In order to perform the dot product at the start and end, $x\left(\lambda \right)$ has to be padded by 2M samples on the left and on the right. A common practice is to pad the ends with zeros; however, other methods, such as padding with the mean value of the signal, linear padding, symmetric padding, periodic extension padding, and fitting polynomial functions, can also be employed [27,68,69]. For the given signal and filter durations, the full-convolution sum can be expressed as

(A2) $$y\left(n\right)=\sum_{\lambda =-M}^{M}\hat{x}\left(\lambda \right)h\left(n-\lambda \right),\;\;\;n=0,\;1,\ldots ,\left(\tilde{N}-1\right),$$

where, $\hat{x}\left(\lambda \right)$ is $x\left(\lambda \right)$ padded with 2M samples on both ends. It is important to observe that the first 2M and the last 2M samples of the output are computed from partial overlaps between $x\left(\lambda \right)$ and $h\left(\lambda \right),$ which are the artifact coefficients in this case. Irrespective of the padding method employed, the outputs computed from the partial overlaps are the inaccurate artifact coefficients.

In practice, full convolution is performed very efficiently in the frequency domain by multiplying the DFTs of $x\left(n\right)$ and $h\left(n\right)$. The filtered output is given by the inverse DFT of the multiplication. The DFTs are computed by augmenting both sequences with zeros to have durations equal to or greater than $\tilde{N}$ for the resulting circular convolution to be identical to the desired linear convolution. The DFTs are computed efficiently using fast Fourier transform (FFT) algorithms [70].

#### Appendix A.1.2. Same-Convolution

This form of convolution is the one used to generate the standard CWT coefficient matrix to ensure that the rows have the same duration as the input. Each row of the coefficient matrix is obtained by performing the same-convolution operation between $x\left(n\right)$ and $h\left(n\right)$. The convolution operation starts by aligning the filter center with the rightmost sample of $x\left(\lambda \right)$ and ends with the alignment of the filter center with the rightmost sample of $x\left(\lambda \right)$. The signal $x\left(\lambda \right)$ is padded with M samples at both ends; therefore, the first M and the last M samples of the output are computed from partial overlaps, which result in artifact coefficients. For the given signal and filter durations, the same-convolution sum can be written as

(A3) $$y\left(n\right)=\sum_{\lambda =-M}^{M}\hat{x}\left(\lambda \right)h\left(n-\lambda \right),\;\;\;n=0,\;1,\ldots ,\left(N-1\right),$$

where, $\hat{x}\left(\lambda \right)$ is $x\left(\lambda \right)$ now padded with M samples on both ends. We refer to the resulting scalogram, which has rows with the same duration as the input signal, as the same (S)-scalogram. The shorter duration same-convolution result can be obtained from full-convolution by cropping out the first M and last M samples. In general, row j of the standard scalogram is generated by same-convolving $x\left(n\right)$ with a wavelet having an effective support $\left[-(S_{j})\left(M\right),(S_{j})\left(M\right)\right]$; therefore, the first and last $(S_{j})\left(M\right)$ coefficients in the row are the artifact coefficients, and the coefficients in between are the accurate coefficients.

#### Appendix A.1.3. Valid Convolution

The valid convolution operation yields coefficients that are accurate and is used to identify the COI in the coefficient matrix. In this form of convolution, the leftmost sample of the centered filter is aligned with the leftmost sample of $x\left(\lambda \right)$ at the start, and the rightmost sample of the centered filter is aligned with the rightmost sample of $x\left(\lambda \right)$ at the end of the convolution. Consequently, the duration of the output is $\left[N-2M\right]$. Valid convolution requires no padding at the ends of $x\left(\lambda \right)$; thus, none of the output samples are computed from partial overlaps resulting in no artifact coefficients. That is, all valid coefficients are computed accurately. Valid convolution can be obtained from same-convolution by cropping the first and last *M* samples and by cropping the first and last 2M samples from full-convolution. For the given signal and filter durations, the valid convolution sum can be written as

(A4) $$y\left(n\right)=\overset{M}{\underset{\lambda =-M}{\sum }}x\left(\lambda \right)h\left(n-\lambda \right),\;\;\;n=0,\;1,\ldots ,\left(N-2M-1\right).$$

As noted in Section 2.1, the COI is the boundary that separates the artifact coefficients from the accurate coefficients in the S-scalogram, the part of the S-scalogram that is inside the COI is the V-scalogram, and the entire S-scalogram with zeroed-out coefficients outside the COI is referred to as the Z-scalogram.

### Appendix A.2. RRS Algorithm

The channel rankings can be computed from the 1-ERPs or the m-ERPs. The RRS algorithm is described in terms of m-ERPs and is applicable to 1-ERPs by setting m = 1. Given C channels, the ranking $R_{m}\left(c\right),\;c=1,2,\ldots ,C$, is such that the channel with the highest multi-class separation is assigned the highest rank=1 and the channel with the smallest is assigned the lowest rank=C. Based on this ranking, channels can be selected by evaluating the performance, starting with the best channel, and adding the next best channels until the performance starts to drop. Alternatively, a subset of channels with the highest ranks can be selected to decrease the computational requirements, especially for dense-electrode arrays. If $L_{mxcb_{i}}$ and $L_{mycb_{i}}$ are the m-ERPs of subject $b_{i},\;i=1,2,\ldots B$, elicited by stimuli $I_{x}$ and $I_{y}$ at channel c, respectively, and $\left|\left|L_{1}-L_{2}\right|\right|$ is the Euclidean distance between $L_{1}$ and $L_{2}$, the interclass separation between the m-ERPs belonging to a pair of classes, $\omega_{x}$ and $\omega_{y}$, at channel c is given by

(A5) $$\rho_{\left(x,y\right)mb_{i}}\left(c\right)=\frac{{\left|\left|\bar{L_{mxcb_{i}}}-\bar{L_{mycb_{i}}}\right|\right|}^{2}}{\sum_{q=1}^{N_{x}}{\left|\left|L_{mxcb_{i}}^{\left(q\right)}-\bar{L_{mxcb_{i}}}\right|\right|}^{2}+\sum_{q=1}^{N_{y}}{\left|\left|L_{mycb_{i}}^{\left(q\right)}-\bar{L_{mycb_{i}}}\right|\right|}^{2}},\;c=1,2,\ldots ,C$$

where $L_{mxcb_{i}}^{\left(q\right)}$ and $L_{mycb_{i}}^{\left(q\right)}$ are the $q^{th}$ m-ERPs; $\bar{L_{mxcb_{i}}}$ and $\bar{L_{mycb_{i}}}$ are the means, and $N_{x}$ and $N_{y}$ are the number of m-ERPs in the $\omega_{x}$ and $\omega_{y}\;m$-ERP ensembles, respectively. Note that the pair $\left(x,y\right)$ takes on the following values:

(A6) $$\;\left(x,y\right)=\left(\omega_{1},\omega_{2}\right),\left(\omega_{1},\omega_{3}\right),\ldots ,\left(\omega_{1},\omega_{J}\right),\left(\omega_{2},\omega_{3}\right),\left(\omega_{2},\omega_{4}\right),\ldots ,\left(\omega_{2},\omega_{J}\right),\ldots ,\left[\omega_{\left(U-1\right)},\omega_{U}\right].$$

The rank $R_{\left(x,y\right)mb_{i}}\left(c\right)$ of $\rho_{\left(x,y\right)mb_{i}}\left(c\right)$ for each subject $b_{i}$ is determined for all possible pairs of x and y. Then, the rank-sum $\left(RS\right)$ of channel c for subject $b_{i}$ across all possible $\left(U\right)\left(U-1\right)/2$ pairs of ERP classes is given by

(A7) $${\left(RS\right)}_{mb_{i}}\left(c\right)=\sum_{x=1}^{U-1}\sum_{y=x+1}^{U}R_{\left(x,y\right)mb_{i}}\left(c\right)$$

The ranking of the channels for subject $b_{i}$ is obtained from the rank of the rank-sum given by

(A8) $$R_{mb_{i}}\left(c\right)=Rank\left\{{\left(RS\right)}_{mb_{i}}\left(c\right)\right\},\;c=1,2,\ldots ,C$$

The ranking of the channels across all B subjects is determined by first summing the ranks to obtain the rank-sum

(A9) $${\left(RS\right)}_{m}\left(c\right)=\overset{S}{\underset{i=1}{\sum }}R_{mb_{i}}\left(c\right)$$

and the final ranking of the channels across the *U* ERP classes and *B* subjects is determined from the rank of the rank-sum given by

(A10) $$R_{m}\left(c\right)=Rank\left\{{\left(RS\right)}_{m}\left(c\right)\right\}.$$

The entire sequence of steps can be summarized as follows:

(A11) $$\left\{Rank\left\{\begin{array}{c} Sum\;across\\ subjects \end{array}\left\{Rank\left\{\begin{array}{c} Sum\;across\\ class\;pairs \end{array}\left\{Rank\left\{\rho_{\left(x,y\right)mb_{i}}\left(c\right)\right\}\right\}\right\}\right\}\right\}\right\}$$

For the special dichotomous (2-class) case, the ranking of the channels for each subject is determined directly from $R_{mb_{i}}\left(c\right)$ which is the ranking of $\rho_{mb_{i}}\left(c\right)$, the interclass separation of the two classes x and y. The ranking across all subjects is obtained from the rank-sum ${\left(RS\right)}_{m}\left(c\right)$ of channel c, which is given by

(A12) $${\left(RS\right)}_{m}\left(c\right)=\overset{S}{\underset{i=1}{\sum }}R_{mb_{i}}\left(c\right)$$

and the final ranking of channel c is given by the rank-of-the rank-sum

(A13) $$R_{m}\left(c\right)=Rank\left\{{\left(RS\right)}_{m}\left(c\right)\right\}.$$

A final comment regarding the application of the RRS algorithm is that the channel rankings are determined by applying the RRS algorithm to the ERPs of only the training set.

## Appendix B

**Table A1:** Classification accuracies of the ERPs of Subject $b_{1}$. Inconsistent results are highlighted in red. The best result for each channel is highlighted in bold font.

Channel	Approach	Classifier					
		SVM	RF	KNN	MLP	CNN-1	CNN-2
P3	1	59.09	77.36	53.77	73.58	76.42	83.74
	2	63.64	83.02	60.38	82.26	83.02	**94.32**
	3	72.73	89.77	69.81	87.74	90.57	---
P1	1	60.91	73.58	56.60	78.49	77.36	81.13
	2	66.36	81.51	50.94	80.57	80.19	92.05
	3	71.82	86.36	63.21	90.57	**92.62**	---
P5	1	58.18	76.42	58.49	71.51	76.04	79.14
	2	59.43	84.91	61.32	72.45	84.91	90.94
	3	69.81	87.74	68.11	86.79	**91.38**	---
Pz	1	57.27	74.91	59.43	73.40	81.89	80.38
	2	56.60	80.38	62.26	76.23	86.79	91.51
	3	66.04	88.64	71.70	84.91	**92.46**	---
P7	1	60.00	76.42	57.55	67.92	75.09	82.45
	2	65.09	85.08	65.66	72.83	82.26	**93.40**
	3	70.81	90.91	73.77	87.36	90.09	---
POz	1	60.91	79.25	54.72	71.89	83.10	84.72
	2	66.04	84.15	59.06	74.34	87.36	92.22
	3	76.23	90.57	66.04	85.85	**94.34**	---
PO2	1	56.36	78.87	53.02	76.42	80.57	81.70
	2	57.55	79.62	60.38	80.06	84.90	94.34
	3	64.16	88.68	68.49	90.38	**95.00**	---
PO1	1	54.55	74.34	50.94	77.36	79.43	79.81
	2	61.32	80.38	56.60	80.38	85.09	**92.45**
	3	71.70	85.85	67.92	88.68	91.89	---

**Table A2:** Classification accuracies of the ERPs of Subject $b_{2}$. Inconsistent results are highlighted in red. The best result for each channel is highlighted in bold font.

Channel	Approach	Classifier					
		SVM	RF	KNN	MLP	CNN-1	CNN-2
PO4	1	55.46	72.73	64.14	72.76	73.47	80.53
	2	60.00	81.82	65.86	83.62	83.67	91.68
	3	72.73	88.18	78.45	90.52	**91.86**	---
F3	1	60.91	75.00	68.79	77.86	76.53	84.21
	2	64.55	82.55	75.35	76.07	81.63	**93.69**
	3	76.36	90.91	79.10	88.95	91.84	---
CP5	1	54.55	81.83	65.52	75.52	75.00	79.31
	2	66.36	85.45	71.55	81.03	79.59	91.58
	3	70.00	**93.64**	82.83	88.08	90.89	---
PO2	1	63.64	72.76	60.52	77.79	79.59	76.21
	2	68.18	79.31	66.38	83.91	83.33	90.42
	3	78.18	86.36	79.83	90.91	**91.67**	---
Oz	1	59.09	73.64	61.21	74.83	76.95	81.52
	2	61.82	85.45	65.52	85.69	88.56	**93.89**
	3	71.82	91.82	77.59	90.51	93.63	---
PO6	1	50.91	80.52	66.38	84.48	77.10	81.13
	2	59.09	85.35	66.67	89.66	83.73	92.03
	3	67.27	91.38	75.35	95.52	**92.75**	---
TP7	1	60.91	75.86	64.66	79.31	73.33	78.64
	2	65.55	82.21	70.69	80.07	79.24	90.86
	3	72.73	90.24	79.14	88.79	**90.84**	---
O1	1	58.72	78.79	63.79	77.41	77.79	83.10
	2	62.93	81.72	70.69	76.72	86.36	92.76
	3	73.07	90.52	74.75	87.27	**94.67**	---

**Table A3:** Classification accuracies of the ERPs of Subject $b_{3}$. Inconsistent results are highlighted in red. The best result for each channel is highlighted in bold font.

Channel	Approach	Classifier					
		SVM	RF	KNN	MLP	CNN-1	CNN-2
O2	1	61.21	72.41	68.10	79.32	78.62	82.66
	2	68.10	79.31	68.97	82.76	81.72	92.69
	3	75.00	92.24	75.52	91.38	**93.10**	---
Oz	1	68.97	72.06	62.93	78.97	71.72	80.21
	2	67.59	86.55	64.66	87.07	72.41	90.83
	3	78.10	**92.68**	72.41	92.83	88.28	---
O1	1	63.28	82.77	62.07	69.83	75.00	81.79
	2	74.14	89.10	65.52	86.21	84.83	94.41
	3	76.72	93.14	75.86	91.38	**95.69**	---
PO6	1	67.24	75.00	64.14	75.86	75.86	82.76
	2	74.14	84.83	62.07	84.48	82.76	**95.69**
	3	78.45	90.41	68.10	92.24	94.83	---
PO4	1	68.10	71.72	61.21	82.76	80.69	80.17
	2	68.97	83.97	65.52	89.66	89.66	90.86
	3	79.48	89.41	71.55	95.52	**96.55**	---
PO2	1	62.07	73.47	61.72	75.17	81.90	78.45
	2	73.28	89.66	65.17	81.72	86.21	89.66
	3	77.55	**94.83**	74.14	90.52	92.24	---
TP7	1	64.66	75.86	67.24	78.62	79.31	79.32
	2	67.24	82.76	69.83	84.83	84.14	93.97
	3	72.41	90.10	77.59	93.97	**97.41**	---
POz	1	68.97	76.55	60.34	80.69	72.07	74.14
	2	62.07	80.34	65.52	83.79	81.04	**92.24**
	3	75.86	89.21	74.14	91.14	90.52	---

**Table A4:** Classification accuracies of the ERPs of Subject $b_{4}$. Inconsistent results are highlighted in red. The best result for each channel is highlighted in bold font.

Channel	Approach	Classifier					
		SVM	RF	KNN	MLP	CNN-1	CNN-2
O2	1	57.55	73.58	52.83	74.15	76.42	81.89
	2	60.75	80.38	58.49	80.19	83.02	**93.77**
	3	69.81	87.74	64.15	88.68	92.51	---
Oz	1	54.72	76.23	52.45	77.36	79.81	80.75
	2	58.46	84.91	59.43	82.08	84.91	92.83
	3	66.98	89.62	63.77	90.56	**93.38**	---
TP7	1	51.89	70.57	56.60	69.81	76.98	80.19
	2	57.36	77.36	61.32	76.42	82.83	**94.34**
	3	63.21	85.85	68.30	83.96	92.89	---
O1	1	50.94	75.66	56.23	77.92	78.30	80.00
	2	53.20	79.06	55.85	80.94	86.79	92.66
	3	63.21	86.79	67.17	89.62	**93.58**	---
F3	1	56.60	79.25	60.38	75.47	81.13	83.96
	2	61.13	83.02	62.26	82.08	87.74	90.23
	3	70.38	89.43	66.04	90.94	**93.77**	---
F1	1	54.72	76.79	51.51	78.11	80.57	82.45
	2	62.26	83.02	54.53	83.92	85.85	95.09
	3	70.19	90.57	65.09	90.19	**96.70**	---
F5	1	58.49	74.34	59.06	70.76	74.53	80.94
	2	60.38	80.19	62.26	79.62	80.02	**93.58**
	3	71.70	88.68	73.58	86.79	92.75	---
CP5	1	53.77	76.42	58.49	77.17	79.25	81.02
	2	53.21	80.94	62.08	83.02	83.40	90.36
	3	64.15	87.74	69.81	89.06	**92.45**	---

**Table A5:** Classification accuracies of the ERPs of Subject $b_{5}$. Inconsistent results are highlighted in red. The best result for each channel is highlighted in bold font.

Channel	Approach	Classifier					
		SVM	RF	KNN	MLP	CNN-1	CNN-2
C2	1	51.89	75.51	51.02	76.73	77.55	81.25
	2	57.55	79.17	59.18	84.69	84.38	**92.71**
	3	64.15	87.50	60.20	89.80	92.63	---
P5	1	52.83	77.55	57.14	79.59	74.49	80.61
	2	58.33	81.63	63.27	80.41	83.47	91.67
	3	65.31	88.54	68.37	87.76	**92.76**	---
Cz	1	58.16	69.80	53.06	74.49	79.59	81.63
	2	61.46	71.63	61.22	78.57	85.71	**92.86**
	3	69.39	86.46	67.35	86.94	91.84	---
C4	1	56.25	74.29	55.31	75.51	79.69	83.68
	2	60.42	79.59	63.64	81.86	84.69	92.92
	3	69.17	85.42	66.12	88.98	**93.88**	---
P3	1	54.79	71.43	58.17	72.45	81.63	83.33
	2	56.88	78.16	64.49	79.39	86.74	**93.88**
	3	70.83	84.69	68.37	85.71	92.45	---
P7	1	56.67	73.47	62.22	76.53	81.08	82.45
	2	58.96	79.39	62.04	80.21	88.78	93.90
	3	68.55	87.76	67.35	89.80	**94.83**	---
C1	1	53.06	74.08	55.10	72.45	75.00	81.63
	2	59.38	77.55	57.14	81.22	79.80	92.86
	3	66.67	85.71	68.98	87.76	**93.20**	---
FCz	1	54.82	71.02	46.94	73.47	76.33	84.08
	2	59.80	74.29	53.06	79.59	84.69	92.22
	3	65.31	86.74	64.29	85.31	**93.82**	---

**Table A6:** Single trial classification accuracies of the 1-ERPs of Subject $b_{3}$. Inconsistent results are highlighted in red. The best result for each channel is highlighted in bold font.

Channel	Approach	Classifier					
		SVM	RF	KNN	MLP	CNN-1	CNN-2
O2	1	44.64	52.73	40.77	54.46	55.10	63.47
	2	50.21	58.16	47.64	57.14	59.23	76.73
	3	55.10	63.54	50.86	65.63	**82.53**	---
Oz	1	45.92	51.38	40.19	53.64	58.16	63.85
	2	47.00	53.10	44.83	57.27	65.31	78.54
	3	54.90	61.40	50.64	66.36	**83.62**	---
O1	1	44.83	52.76	40.38	54.14	52.79	67.08
	2	49.79	58.62	42.94	59.09	61.22	75.71
	3	55.17	63.79	50.43	68.42	**81.63**	---
PO6	1	42.42	49.06	43.40	54.72	51.07	61.88
	2	48.93	56.60	45.28	59.48	57.14	79.30
	3	53.27	60.38	50.09	67.24	**80.41**	---
PO4	1	42.31	47.17	40.56	55.66	54.72	61.23
	2	41.53	54.63	40.40	59.43	63.83	**80.61**
	3	53.96	61.32	51.89	66.57	77.55	---
PO2	1	42.59	44.45	40.74	52.24	50.86	69.79
	2	43.08	50.96	43.70	55.66	64.29	**80.18**
	3	54.72	57.55	51.13	64.15	79.80	---
TP7	1	45.28	51.85	40.94	51.85	55.32	61.43
	2	48.15	51.67	48.15	56.44	65.96	78.82
	3	56.60	62.26	52.83	63.77	**80.83**	---
POz	1	43.43	50.00	40.03	50.56	51.50	66.15
	2	49.23	57.41	47.96	59.26	63.86	72.73
	3	54.34	60.18	54.19	62.45	**79.59**	---
